# Efficacy of the Web-Based Swedish Individualized Active Communication Education (I-ACE) Program in First-Time Hearing Aid Users: Randomized Controlled Trial

**DOI:** 10.2196/71975

**Published:** 2025-07-25

**Authors:** Louise Werther, Elisabet Sundewall Thorén, Jonas Brännström, Gerhard Andersson, Marie Öberg

**Affiliations:** 1 Department of Otorhinolaryngology in Östergötland and Department of Biomedical and Clinical Sciences, Linköping University, University Hospital, Linköping, 58185, Sweden, 46 0724638793; 2 Department of Otorhinolaryngology, Head and Neck Surgery, Audiology Clinic, Skåne University Hospital, Lund, Sweden; 3 Department of Clinical Science, Logopedics, Phoniatrics and Audiology, Lund University, Lund, Sweden; 4 Department of Clinical Neuroscience, Karolinska Institutet, Stockholm, Sweden; 5 Department of Behavioural Sciences and Learning, Linköping University, Linköping, Sweden

**Keywords:** I-ACE, ACE, eHealth, hearing loss, aural rehabilitation, communication strategies, active communication education

## Abstract

**Background:**

Hearing loss is estimated to affect more than 20% of the global population. Hearing aid fitting is a common intervention in audiological rehabilitation; however, there are still those who struggle with remaining communication difficulties that require additional intervention. The group rehabilitation program Active Communication Education (ACE) has been shown to be an effective alternative for addressing these remaining difficulties. To increase accessibility, the ACE program was modified to an individual version. The effects of the Individualized Active Communication Education (I-ACE) are yet to be explored in a randomized controlled trial.

**Objective:**

This study aims to explore the effects of the internet-based Swedish I-ACE program on the use of communication strategies, self-perceived hearing difficulties, and the emotional effects of hearing loss, as well as whether these effects persist long-term.

**Methods:**

First-time hearing aid users with 6‐12 months of experience with hearing aids were invited to participate in a randomized controlled trial. The participants were allocated to either the Swedish I-ACE intervention program or the delayed intervention control group. The 5 chapters of the I-ACE were delivered over a 5-week period through the nationally available online health portal for the public health system, 1177.se. Each week, feedback was provided through the platform by a clinician. The efficacy of the I-ACE was determined by change of the primary outcome of emotional consequences and acceptance of hearing loss, and use of communication strategies. The secondary outcome measures included perceived hearing difficulties, hearing aid efficacy, and intervention efficacy. Participants completed the self-assessed outcome measures at baseline (T0), post intervention (T1), and 6 months post intervention (T2) through the platform.

**Results:**

A total of 57 participants (I-ACE, n=29 and delayed intervention control, n=28) were included in the analyses. Compared with the control group, the Swedish I-ACE improved the emotional consequences and acceptance of hearing loss, as well as use of communication strategies (the Communication and Acceptance Scale, *F*_1, 54_=7.26, *P*=.01 and the Communication Strategies Scale, *F*_1, 53_=6.35, *P*=.02). There were no differences in perceived hearing difficulties or hearing aid efficacy between the two groups. All outcomes remained stable at the 6-month follow-up.

**Conclusions:**

The results suggest that the I-ACE program can be an effective alternative for reducing emotional consequences of hearing loss and increasing the use of communication strategies to reduce remaining communication difficulties.

## Introduction

It is estimated that over 20% of the global population is affected by hearing loss, with 5.5% facing life with a disabling hearing impairment [1]. The negative consequences of untreated hearing loss have been widely documented, including increased social isolation [2], poorer quality of life [3], and decreased cognitive ability [4]. It is common to be offered hearing aid fitting as audiological rehabilitation when seeking help due to hearing loss. Although many of those who have been fitted with hearing aids express being satisfied with their hearing aids and use them regularly [5], some still struggle with the remaining communication difficulties associated with hearing loss [6].

For those struggling with remaining communication difficulties, additional interventions may be necessary. The Active Communication Education (ACE) program is a group rehabilitation program designed to address communication difficulties associated with hearing loss [7]. It aims to facilitate more effective communication for individuals with hearing loss, equipping them with strategies to integrate and use in their daily lives. The ACE is composed of a series of modules, each of which addresses a specific theme related to challenging communication situations. The content of these modules is discussed in 2-hour weekly group sessions over a 5-week period. The program has been shown to be effective in reducing communication limitations and participation restrictions resulting from hearing loss [8]. However, similar effects were found for the study’s control group, which received a social program, and the differences between the groups were statistically nonsignificant. The ACE program was later modified into a version that could be completed individually to increase accessibility for individuals who may have practical difficulties attending or prefer not to participate in group settings. This new version, the Individualized Active Communication Education (I-ACE) program, allows for independent completion of the program’s activities and materials. Similar to the group-delivered ACE, the I-ACE has been shown to reduce participation restrictions and activity limitations [9].

Both the ACE and I-ACE programs have been translated and modified for the Swedish population. The Swedish ACE program has been evaluated in several studies, which have demonstrated its effectiveness in reducing participation restrictions and activity limitations, as well as increasing the use of communication strategies [10-12]. The effects of the Swedish I-ACE program have only been evaluated in pilot studies. In these studies, effects similar to those for the Swedish ACE were found for participation restriction and activity limitation, as well as communication strategies [13,14].

While the ACE program is an onsite group intervention, previous research has shown that eHealth can be used to provide audiological rehabilitation with positive results [15,16]. To facilitate the use and accessibility of the Swedish I-ACE program in audiological rehabilitation, the program was adapted for the online health portal for the public health care system, 1177.se. This platform is nationally available to residents and health care professionals in Sweden, providing secure communication and interaction. In a small previous study (n=12), participants’ experiences with the material as well as the feasibility of providing the Swedish I-ACE via the Support and Treatment platform of 1177.se were assessed [17]. The participants of the study found the material informative, relevant, and comprehensible. Furthermore, the results showed that it was feasible to deliver the program through the new platform, with few participants reporting difficulties using the platform and accessing the program. However, the efficacy of the Swedish I-ACE program has yet to be explored.

The aim of this study was to explore the short-term effects of the Swedish I-ACE program in first-time hearing aid users compared with a delayed intervention control group in regard to the use of communication strategies, self-perceived hearing difficulties, and the emotional effects of hearing loss. Furthermore, we aimed to explore whether any effects persisted long-term.

## Methods

### Trial Design

#### Overview

This study was registered at ClinicalTrials.gov (NCT05815667). It used a 2-arm, delayed intervention, randomized controlled trial design. With an allocation ratio of 1:1, participants were randomized to the intervention group receiving the Swedish I-ACE, or the delayed intervention control group. The control group received the same intervention after the intervention group had completed the Swedish I-ACE.

#### Recruitment

First-time hearing aid users who were fitted with hearing aids 6‐12 months before at a clinic in the county of Östergötland, Sweden, were invited to participate in the study during January and February 2023. They were sent information about the study via mail, including informed consent forms and a few questions assessing computer experience for eligibility. Those who consented to participate in the study were called for a short telephone interview to reassess eligibility and provide an opportunity to ask any remaining questions. The inclusion criteria were (1) first-time users of hearing aids fitted 6‐12 months before; (2) adults, aged ≥20 years; (3) those who were able to read and write in Swedish; (4) those with computer experience and access to a computer, tablet, or smartphone; and (5) those who had not participated in the Swedish ACE group rehabilitation program. Participants who did not meet the inclusion criteria were excluded prior to randomization.

#### Randomization

The participants who met the inclusion criteria were randomly allocated to the intervention or control group. The randomization was performed by a clinician independent from the research team, using a computer-generated block randomization schedule. The participants of the allocated groups were then assigned to an attending clinician using simple randomization.

#### Swedish I-ACE

**Figure 1.**
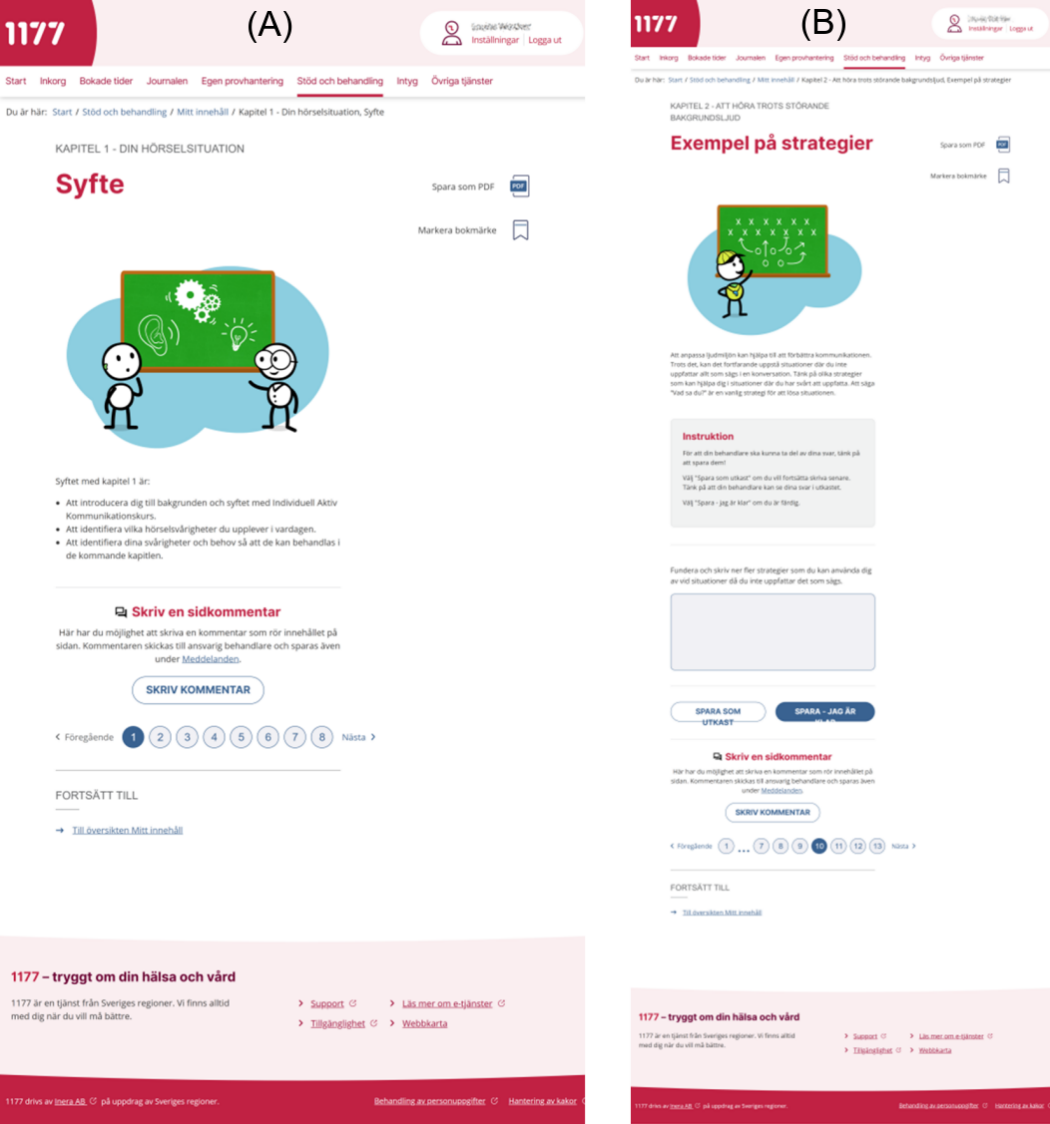
Screenshots of the Swedish I-ACE program on 1177.se: (**A**) example of a chapter start page that explains the purpose of the chapter and (**B**) example of a reflection task for the participant to complete. I-ACE: Individualized Active Communication Education.

The Swedish I-ACE is an interactive intervention program aimed at increasing the use of communication strategies and reducing communication difficulties as a result of hearing loss. It consists of 5 chapters scheduled to be completed 1 per week for the duration of 5 weeks. Each chapter covers a theme, addressing topics such as communication in background noise, the ear and hearing, and communication strategies. The chapters contain information related to the theme of the specific chapter, reflection tasks, and assignments to complete (see Figure 1). Participants complete their assignments through the platform, writing their reflections and answers for the clinician to read. The content of the Swedish I-ACE, as well as the themes and aims of the chapters, is described further in a previous feasibility study [17]. Some of the assignments of the program are designed to include a communication partner and are optional to complete. At the end of each week, feedback is provided by the attending clinician on the completed assignments before the next chapter is assigned.

### Outcome Measures

#### Primary Outcome Measure

The participants were asked to complete a set of patient-reported outcome measures (PROMs) at baseline (T0), post intervention (T1), and 6 months post intervention (T2). These were administered digitally through the Support and Treatment platform.

The primary outcome measure was the recently developed and validated Communication and Acceptance Scale (CAS) [18]. The scale was specifically developed for the evaluation of the ACE program. It measures emotional consequences and acceptance of hearing loss, as well as the use of communication strategies. The CAS consists of 18 items scored on a 5-point scale ranging from almost never (1) to almost always (5), with half of the items reverse scored. The total score ranges from 18 to 90 points, with lower scores indicating greater emotional consequences of hearing loss and less acceptance and use of communication strategies. The items are divided into 5 subscales: emotional consequences (8 items), verbal communication strategies (3 items), confirmation strategies (2 items), hearing knowledge (1 item), and hearing loss and acceptance (4 items). An example of an item is “I avoid communication with others due to my hearing difficulties.”

#### Secondary Outcome Measures

The secondary outcome measures were the Communication Strategies Scale (CSS) [19], the Hearing Handicap Inventory for the Elderly (HHIE) [20], the International Outcome Inventory for Hearing Aids (IOI-HA) [21], and the International Outcome Inventory for Alternative Interventions (IOI-AI) [22].

The CSS is a 25-item scale measuring the use of various types of communication strategies. The items are scored on a 5-point scale ranging from almost never (1) to almost always (5), and divided into 3 subscales: maladaptive (9 items, reverse scoring), verbal (8 items), and nonverbal strategies (8 items). The total score ranges from 25 to 125 points, with a lower score indicating less use of communication strategies. A validated Swedish version was used [23]. An example of an item is “In difficult listening situations, I try to position myself so that I can hear as well as possible.”

The HHIE is a 25-item scale measuring perceived hearing difficulties. The items are rated using 3 alternatives, “yes” (4), “sometimes” (2), and “no” (0), and are divided into 2 subscales, emotional (13 items) and social (12 items) consequences. The total score ranges from 0 to 100 points, with a lower score indicating less perceived hearing difficulties. A validated Swedish version was used [24]. An example of an item is “Does a hearing problem cause you to visit friends, relatives, or neighbors less often than you would like?”

The IOI-HA is a 7-item scale measuring hearing aid efficacy. Each item is rated on a 5-point scale, with the total score ranging from 7 to 35 points. The items are divided into 2 subscales, one encompassing satisfaction with the hearing aids (4 items) and the other with the residual problems (3 items). A higher score is indicative of a better outcome. A validated Swedish version was used [24]. An example of an item is “Think about the situation where you most wanted to hear better, before you got your present hearing aid(s). Over the past two weeks, how much has the hearing aid helped in that situation?”

The IOI-AI extension of the IOI is a 7-item scale measuring the efficacy of an intervention. Like the IOI-HA, each item is rated on a 5-point scale, with the total score ranging from 7 to 35 points. A higher score is indicative of a better outcome. A Swedish translation was used [10]. An example of an item is “Think about how much you used the strategies you learnt in the Active Communication Education over the past 2 weeks. On an average day, how many hours did you use them?”

### Procedure

The participants were assigned the Swedish I-ACE through the Support and Treatment platform. Upon the initial login, they were prompted to complete the T0 PROMs through the platform before accessing the intervention program. The participants were assigned a chapter to complete each week and received individual written feedback on their completed assignments at the end of each week. This was provided through the platform prior to being assigned the next chapter.

In this study, 3 clinicians provided the intervention, each responsible for a number of participants. This included providing feedback and assigning new chapters, answering any questions participants might have, as well as monitoring the participants’ progress and contacting those who had not completed their assignment as scheduled. To ensure that the clinicians provided similar treatment, they followed a treatment protocol and attended weekly meetings to discuss any arisen issues and progress.

A total of 6 weeks after the intervention group began the program, the control group received the same intervention. The Swedish I-ACE is scheduled to be completed in 5 weeks, leaving a 1-week buffer for any participants needing extra time to complete a chapter.

The participants were asked to complete the T1 PROMs upon completion of the intervention and the T2 PROMs at 6 months after completing the intervention. If the participant did not complete the PROMs, an initial reminder was sent out after a week, followed by a second reminder a few days before the 2-week deadline.

### Statistical Analysis

Statistical analyses were performed using SPSS (version 29.0.1.1; IBM Corp). The statistical tests were 2-tailed with α set to .05. Participants who did not complete the intervention or follow-up assessment measures were included in the analysis on an intention-to-treat basis with imputation of the missing data. An analysis of missing data determined the data to be missing completely at random (MCAR) and addressed using multiple imputation with the Markov chain Monte Carlo method [25].

Differences between groups in participant characteristics and outcome measures at T0 were examined using 2-tailed *t* tests for numeric variables and *χ*^2^ tests for categorical variables.

The primary outcome analysis measured differences in outcome measures at T1. Analysis of covariance (ANCOVA) was used to determine outcome differences between the intervention and control groups at T1, using the results at T0 as covariate. The prerequisites for ANCOVA were met. The between-groups effect sizes were calculated using the adjusted means from the ANCOVAs.

Paired sample *t* tests were used to compare differences between T1 and T2 to evaluate the long-term effects of the intervention. Since the control group had received treatment at T2 and was therefore no longer passive, the analyses were conducted for the treatment group only.

### Sample Size Calculation

The sample size was calculated based on the observed mean differences and the SDs of the CAS when used in clinical practice to evaluate the Swedish ACE program. Using these data, assuming an intergroup difference of −6.0 points (SD 9.0) with a significance level of .05 and power of .80, the calculation resulted in a sample size of 36 participants per group.

### Ethical Considerations

The study was approved in advance by the Swedish Ethical Review Authority (file 2022-01982-02). Informed written consent was provided by all participants before enrollment in the study. Participants were informed that they could withdraw from the study at any time and that there was no compensation for participation. The data were deidentified.

The use of a delayed intervention control group was carefully considered when designing the study. Participants of the control group would have to wait 6 weeks to receive the intervention, which is well within the Swedish 90-day guarantee of care. Participants were also informed in the invitation to participate in the study that there would be 2 starting dates of the intervention. It was however deemed unethical to withhold a potentially beneficial intervention for 6 months to ensure a passive control group for the long-term follow-up.

## Results

### Overview

**Figure 2.**
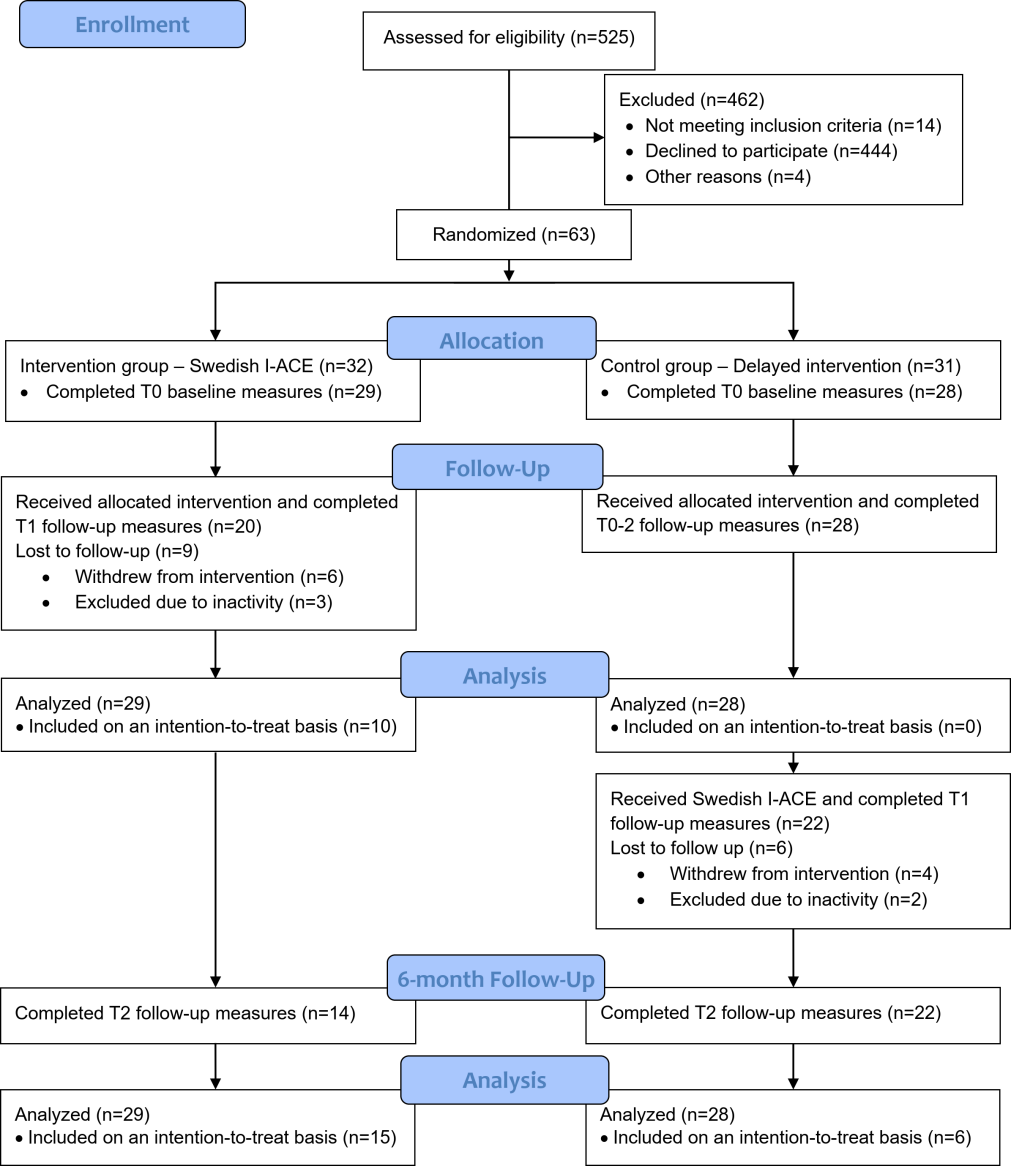
The CONSORT (Consolidated Standards of Reporting Trials) flow diagram of the study. I-ACE: Individualized Active Communication Education.

A total of 525 individuals were invited to participate in the study, of which 81 provided informed consent to participate, as well as the form assessing computer experience. Of these, 14 participants did not meet the inclusion criteria, and 4 participants had expressed other needs for intervention during the telephone assessment and were excluded before randomization. Figure 2 shows that during randomization, 32 participants were allocated to the intervention group, I-ACE, and 31 participants were allocated to the control group. A total of 3 participants in each group were excluded due to not completing the baseline measures, leaving 29 and 28 participants in the groups included in the analysis.

### Participant Characteristics

The characteristics of the participants included in the analysis are described in Table 1. The mean age of the total sample (N=57) was 70 years (SD 12.0; range 25‐91). The gender distribution was found to be even, with 30 (53%) participants identifying as male and 27 (47%) as female. The participants’ mean hearing thresholds for pure tone audiometry were 33.2 dB HL (SD 8.4) for the right ear and 37.0 dB HL (SD 12.2) for the left. Almost all of the participants used the internet several times per day or daily (54/57, 95%), and more than half of them (34/57, 60%) had a postsecondary education. The majority of participants were fitted with 2 hearing aids (51/57, 90%), and it was most common to use the hearing aids more than 8 hours/day (26/57, 46%). The groups were well matched, as presented in Table 1, and there were no statistically significant differences (*P*>.05) between the groups for any of the demographics presented in Table 1.

**Table 1.** Participant characteristics.

Characteristics		Intervention group (n=29)	Control group (n=28)
Age (years)			
Mean (SD)		70 (7.9)	70 (15.2)
Range		50‐83	25‐91
Gender, n (%)			
Male		15 (51.7)	15 (53.6)
Female		14 (48.3)	13 (46.4)
PTA4^a^ (dB HL), mean (SD)			
Right ear		34.3 (6.9)	31.9 (9.6)
Left ear		35.0 (10.6)	39.0 (13.4)
Highest level of education, n (%)			
Presecondary		4 (13.8)	4 (14.3)
Secondary		7 (24.1)	5 (17.9)
Postsecondary		17 (58.6)	17 (60.7)
Missing		1 (3.5)	2 (7.1)
Internet use, n (%)			
Several times per day		17 (58.6)	16 (57.1)
Every day		11 (37.9)	10 (35.7)
Every week		1 (3.4)	2 (7.2)
Hearing aids, n (%)			
Unilateral		2 (6.9)	4 (14.3)
Bilateral		27 (93.1)	24 (85.7)
Hearing aid use, n (%)			
<1 hour/day		5 (17.2)	2 (7.1)
1‐4 hours/day		6 (20.7)	7 (25)
4‐8 hours/day		7 (24.1)	4 (14.3)
>8 hours/day		11 (37.9)	15 (53.6)

a PTA4: Pure tone average for the frequencies 500, 1000, 2000, and 4000 Hz.

### Outcomes

#### Overview

Table 2 reports the means and SDs for both groups at T0 and T1. Furthermore, the results of the ANCOVAs, along with their respective effect sizes, are presented. There were no statistically significant differences between the groups at T0 for the CAS (*t*_55_=−0.58; *P*=.56), CSS (*t*_55_=0.20; *P*=.84), HHIE (*t*_55_=0.52; *P*=.60), or IOI-HA (*t*_55_=−0.65; *P*=.52).

**Table 2.** Mean (SD) at baseline (T0) and post intervention (T1) for intervention and control group, and results of the analyses of covariance (ANCOVA).

		Intervention (n=29)		Control (n=28)		Main effect of group		
		T0, mean (SD)	T1, mean (SD)	T0, mean (SD)	T1, mean (SD)	*F* test (*df*)	*P* value	Cohen *d* (95% CI)^a^
CAS^b^								
Total		61.24 (10.66)	68.66 (8.95)	62.71 (8.44)	64.49 (7.98)	7.26 (1,54)	.01	0.68 (0.14 to 1.21)
Emotional consequences		28.41 (6.63)	31.40 (5.28)	29.36 (5.44)	30.16 (4.68)	2.90 (1,54)	.11	0.42 (−0.10 to 0.95)
Verbal communication strategies		10.34 (2.36)	11.04 (1.99)	9.86 (2.27)	10.31 (2.74)	1.03 (1,54)	.42	0.21 (−0.31 to 0.73)
Confirmation strategies		5.76 (1.38)	6.50 (1.69)	6.07 (1.12)	5.81 (1.35)	4.47 (1,54)	.054	0.51 (−0.02 to 1.03)
Hearing knowledge		3.03 (1.09)	4.04 (0.63)	3.18 (0.82)	3.19 (0.83)	22.41 (1,54)	<.001	1.07 (0.51 to 1.62)
Hearing loss and acceptance		13.69 (2.57)	15.68 (2.01)	14.25 (2.50)	15.01 (2.13)	3.54 (1,54)	.07	0.49 (−0.04 to 1.02)
CSS**^c^**^,d^								
Total		82.61 (10.71)	87.72 (11.45)	83.14 (9.15)	82.81 (8.51)	6.35 (1,53)	.02	0.64 (0.10 to 1.17)
Maladaptive		39.04 (4.05)	37.87 (3.04)	39.32 (2.89)	38.69 (3.55)	0.87 (1,53)	.39	−0.22 (−0.75 to 0.30)
Verbal		20.18 (5.46)	22.80 (4.93)	19.36 (4.58)	19.62 (5.26)	5.72 (1,53)	.03	0.60 (0.07 to 1.14)
Nonverbal		23.39 (6.84)	27.05 (6.15)	24.46 (6.26)	24.50 (6.62)	5.97 (1,53)	.02	0.63 (0.09 to 1.16)
HHIE^d,e^								
Total		25.29 (13.90)	22.46 (9.53)	24.79 (13.27)	24.30 (11.71)	1.00 (1,53)	.35	−0.25 (−0.78 to 0.28)
Emotional		12.93 (8.80)	10.14 (5.22)	11.64 (7.52)	11.07 (6.89)	1.34 (1,53)	.16	−0.30 (−0.83 to 0.22)
Social		12.36 (5.91)	12.31 (5.48)	13.14 (7.29)	13.23 (5.84)	0.32 (1,53)	.60	−0.10 (−0.63 to 0.42)
IOI−HA^f^								
Total		26.66 (4.62)	26.69 (3.91)	27.43 (4.33)	27.94 (3.88)	1.31 (1,54)	.30	−0.27 (−0.79 to 0.25)
Satisfaction with hearing aids		14.83 (3.59)	15.42 (3.05)	16.00 (3.16)	16.36 (2.87)	0.20 (1,54)	.75	−0.07 (−0.59 to 0.45)
Residual problems		11.83 (2.14)	11.27 (1.92)	11.43 (2.17)	11.58 (1.71)	1.47 (1,54)	.24	−0.31 (−0.84 to 0.21)

a Effect size is calculated from the ANCOVAs’ adjusted means.

b CAS: Communication and Acceptance Scale.

c CSS: Communication Strategies Scale.

d Intervention group n=28, 1 case excluded due to extreme outlier.

e HHIE: Hearing Handicap Inventory for the Elderly.

f IOI-HA: International Outcome Inventory for Hearing Aids.

#### Primary Outcome

For the primary outcome of emotional consequences and acceptance of hearing loss, and the use of communication strategies as measured by the CAS, the intervention group increased their total score significantly compared to the control group at T1 (*F*_1, 54_=7.26; *P*=.01). This difference in outcome was of a medium effect size (Cohen *d*=0.68, 95% CI 0.14‐1.21). For the subscales, no statistically significant differences were found between groups, except for hearing knowledge (*F*_1, 54_=22.41; *P*<.001) which also demonstrated a large effect size (Cohen *d*=1.07, 95% CI 0.50‐1.62). The results of the analyses of the CAS subscales are presented further in Table 2.

#### Secondary Outcomes

A statistically significant difference in the use of communication strategies (*F*_1, 53_=6.35, *P*=.02) at T1, as indicated by change in CSS total score, was found with a medium effect (Cohen *d*=0.64, 95% CI 0.10‐1.17). The same was found for the verbal and nonverbal subscales (see Table 2). However, no statistically significant differences were identified for either perceived hearing difficulties as measured by the HHIE (*F*_1, 53_=1.00, *P*=.35) or hearing aid efficacy as measured by the IOI-HA (*F*_1, 54_=1.31, *P*=.30). Further results of the analyses of the secondary outcome subscales are presented in Table 2.

#### Long-Term Effects at 6 Months

Table 3 reports the results from the paired samples *t* tests for the intervention group between T1 and T2. For the primary outcome measure, the CAS, there was no change in score between T1 and T2 for the intervention group (*t*_28_=1.03; *P*=.30). For the subscales, the same results were found except for verbal communication strategies, which had increased (*t*_28_=2.36; *P*=.02).

**Table 3.** Results of the paired samples *t* tests for the intervention group (n=29) between post intervention (T1) and 6 months post intervention (T2).

		Mean difference T1 to T2 (95% CI)		Paired *t* test (*df*)		*P* value
CAS^a^						
Total		1.83 (−2.18 to 5.83)		1.03 (28)		.30
Emotional consequences		0.14 (−2.31 to 2.58)		0.13 (28)		.90
Verbal communication strategies		1.09 (0.11 to 2.07)		2.36 (28)		.02
Confirmation strategies		−0.06 (−1.03 to 0.92)		−0.13 (28)		.90
Hearing knowledge		0.17 (−0.26 to 0.60)		1.01 (28)		.32
Hearing loss and acceptance		0.48 (−0.61 to 1.57)		1.00 (28)		.32
CSS^b^^,^^c^						
Total		3.40 (−1.12 to 7.92)		1.84 (27)		.07
Maladaptive		1.27 (−0.24 to 2.78)		1.82 (27)		.08
Verbal		5.44 (2.83 to 8.04)		4.77 (27)		<.001
Nonverbal		1.19 (−1.67 to 4.04)		1.03 (27)		.30
HHIE^b^^,^^d^						
Total		−1.99 (−5.77 to 1.80)		1.21 (27)		.23
Emotional		−1.27 (−3.88 to 1.34)		1.22 (27)		.22
Social		−0.71 (−2.92 to 1.50)		0.66 (27)		.52
IOI-HA^e^						
Total		0.61 (−0.89 to 2.10)		0.96 (28)		.34
Satisfaction with hearing aids		0.12 (−0.98 to 1.22)		0.23 (28)		.82
Residual problems		0.49 (−0.54 to 1.52)		1.12 (28)		.26
IOI-AI^f^						
Total		0.61 (−0.88 to 2.09)		0.81 (28)		.42

a CAS: Communication and Acceptance Scale.

b One extreme outlier excluded from analysis

c CSS: Communication Strategies Scale.

d HHIE: Hearing Handicap Inventory for the Elderly.

e IOI-HA: International Outcome Inventory for Hearing Aids.

f IOI-AI: International Outcome Inventory for Alternative Interventions.

For the secondary outcome measures, the use of verbal communication strategies (CSS) had increased 6 months post intervention (*t*_27_=4.77; *P*<.001). There was no change in perceived hearing difficulties (HHIE) (*t*_27_=1.21, *P*=.23) or hearing aid efficacy (IOI-HA; *t*_28_=0.96, *P*=.34). The results for the subscales are presented in Table 3.

**Figure 3.**
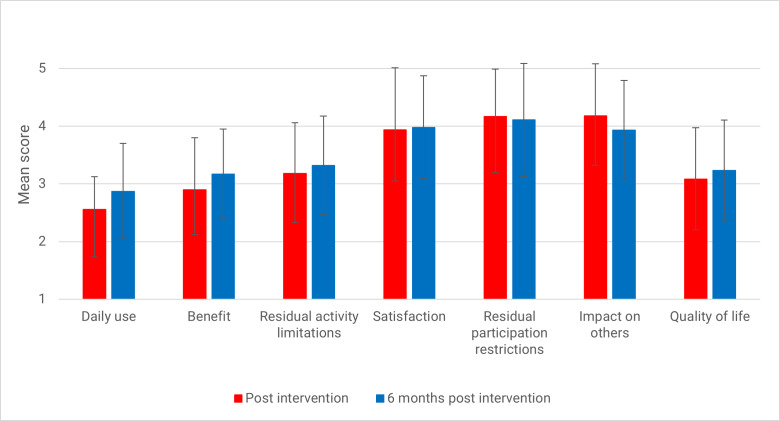
Means and SDs per item of the International Outcome Inventory for Alternative Interventions (IOI-AI). A higher score is indicative of a better outcome.

There was no change in intervention efficacy (IOI-AI) from T1 to T2 (*F*_1, 56_=0.44; *P*=.44). Figure 3 shows the mean score and SDs of each item of the IOI-AI at T1 (post intervention) and T2 (6 mo post intervention).

### Attrition

Overall, 67% (42/63) of participants completed the I-ACE program, leaving an attrition of 33% (21/63). The attrition did not differ (*χ*^2^=1.20; *P*=.27) between the intervention (38%, 12/32) and control group (30%, 9/31).

When comparing the baseline characteristics between those who completed the I-ACE and those who did not, there were no statistically significant differences except for age (*t*_55_=2.31; *P*=.02). The participants who did not complete the I-ACE program were younger (mean 64.2, SD 9.0 y) than those who did (mean 72.0, SD 16.5 y).

## Discussion

### Principal Findings

This study aimed to explore the effects of the Swedish I-ACE program on the use of communication strategies and on self-perceived hearing difficulties and effects of hearing loss, both short- and long-term.

For the primary outcome measure, the CAS, the intervention group significantly improved its score compared to the control group. Although the change in total score showed a statistically significant difference between the groups, the same was only found for 1 of the 5 subscales. The CAS measures a variety of dimensions with some subscales containing few items. The intervention group had increased scores on all subscales at T1, contributing to the increase in total score. However, because some subscales contain few items, the increase for that subscale may be small. When comparing the verbal communication strategies subscale for the CAS and CSS, the former contains 3 items compared to 8. For the CAS, there was no statistically significant difference between the groups for this subscale, whereas there was for the corresponding subscale of the CSS.

For other subscales, that is, emotional consequences, the nonsignificant difference between groups is consistent with the corresponding subscale of the HHIE.

The intervention group significantly increased the use of communication strategies compared with the control group. Similar results have been reported in previous studies exploring the effects of the ACE program [11,12]. The use of both verbal and nonverbal communication strategies increased post intervention; however, no change in the use of maladaptive communication strategies was found. These results are similar to those reported in previous studies of the ACE program [11,12], as well as other internet-based interventions that include information on communication strategies [26,27]. In assessing the baseline scores of this study, the participants did not report using maladaptive communication strategies to any great extent before the intervention, making further improvement difficult.

Regarding self-perceived hearing difficulties, no differences were found between the groups post intervention. Studying the results of the baseline data from the HHIE, the scores were fairly low in comparison to those reported in other studies of group-based and internet-based interventions [12,26,28]. This might be explained by the low mean PTA of the population, which corresponds to mild hearing loss [1]. Some of the mentioned previous studies excluded participants with HHIE scores lower than 20 points, which could be one explanation for the discrepancy. With these low baseline scores, indicating initially low self-perceived hearing difficulties, it might prove difficult to achieve an improvement.

There were no changes in hearing aid efficacy (IOI-HA) after the intervention. No intervention effort specifically targets hearing aids in the I-ACE program; however, participants are informed of communication strategies to facilitate communication. This would also be beneficial during hearing aid use, which together with the potential increased use of hearing aids, was thought to potentially affect hearing aid efficacy in participants post intervention. As for the intervention efficacy (IOI-AI), there was no change between post intervention and at the 6-month follow-up. At post intervention, the participants in this study scored similarly to participants in previous studies of the ACE and I-ACE programs, making them comparable. However, at the 6-month follow-up, they tended to score higher than at post intervention, as opposed to other studies of the ACE program in which participants tended to score lower over time [9,11,12,29].

When comparing the results post intervention and at 6 months post intervention, there was generally no change in scores. This suggests that the effects of the I-ACE are stable over time. In general, the group means tended to improve from post intervention to 6 months post intervention; however, this improvement was statistically significant only for the verbal communication strategies subscales of the CAS and the CSS. Changing the use of communication strategies is a behavioral change that can take time and therefore also result in a delayed intervention effect. However, it is important to note that these results were not compared to a control group.

Participants who did not complete the I-ACE program were younger than those who did. Among the noncompleters, there was a lower proportion of participants who were retired (50%, 8/16) compared to the completers (85%, 35/41). This could indicate that those who have employment may find it difficult to take the time to engage in the intervention. The I-ACE program is more flexible for the participant compared to the group ACE program, leaving the participant to complete the assignments according to their own schedule. Some participants might however need more flexibility in terms of time allowed to complete each chapter.

### Limitations

A limitation of this study was the small sample size. Although many were assessed as eligible for participation, only 15% (81/525) of those invited consented to participate in the study. A potential explanation for the low consent rate is that those invited to participate in the study had recently undergone audiological rehabilitation through hearing aid fitting and might not have felt the need for further intervention at the time. Those who consented to participate may have been more motivated to complete the intervention, which in turn could have influenced the results. However, 33% (21/63) of the total number of participants did not complete the intervention program, which argues against participants being exceedingly motivated.

Another limitation of the study was that the control group was not passive at 6 months post intervention, resulting in the inability to compare long-term effects between groups. The decision to provide the control group with the intervention was made owing to ethical considerations. It was deemed unethical to withhold a potentially beneficial intervention from the participants for the required time to enable the control group to remain passive.

### Conclusions

The results of this study suggest that the I-ACE program increases the use of communication strategies and that participants continue to demonstrate this increase 6 months after the intervention. Therefore, the I-ACE program is suggested as an option for individuals with hearing loss who are in need of additional intervention and have difficulty with participating or prefer not to participate in group settings. For future directions, a larger study including experienced hearing aid users and varying degrees of hearing loss is suggested, enabling analyses to determine whether any subgroups benefit particularly from the I-ACE program.

## Supplementary material

10.2196/71975 Checklist 1 CONSORT-eHEALTH checklist (V 1.6.1).

## Abbreviations

ACE: Active Communication Education
ANCOVA: analysis of covariance
CAS: Communication and Acceptance Scale
CSS: Communication Strategies Scale
HHIE: Hearing Handicap Inventory for the Elderly
I-ACE: Individualized Active Communication Education
IOI-AI: International Outcome Inventory for Alternative Interventions
IOI-HA: International Outcome Inventory for Hearing Aids
MCAR: missing completely at random
PROM: patient-reported outcome measure
